# A Systematic Review of the Impact of Fat Quantity and Fatty Acid Composition on Postprandial Vascular Function in Healthy Adults and Patients at Risk of Cardiovascular Disease

**DOI:** 10.1016/j.cdnut.2023.102025

**Published:** 2023-10-30

**Authors:** Hannah F. Kienēs, Sarah Egert

**Affiliations:** Institute of Nutritional and Food Sciences, Nutritional Physiology, University of Bonn, Bonn, Germany

**Keywords:** fat quantity, SFA, MUFA, PUFA, CVD, postprandial, vascular function, FMD, PWV, AIx

## Abstract

Atherosclerosis is a key risk factor for developing cardiovascular diseases (CVDs). Flow-mediated dilation (FMD), which reflects vascular reactivity, as well as pulse wave velocity (PWV) and augmentation index (AIx), both markers of arterial stiffness, have emerged as noninvasive, subclinical atherosclerotic markers for the early stages of altered vascular function. In addition to the long-term effects of diet, postprandial processes have been identified as important determinants of CVD risk, and evidence suggests an acute effect of fat quantity and fatty acid (FA) composition on vascular function. However, robust analyses of this association are lacking, especially concerning parameters of arterial stiffness. Therefore, we carried out a systematic literature search in PubMed, Scopus, and the Cochrane Library to investigate the impact of fat quantity and FA composition of meals on postprandial vascular function. Postprandial studies measuring FMD, PWV, and/or AIx in healthy adults and subjects with increased CVD risk (e.g., those with hypercholesterolemia or metabolic syndrome) were analyzed. In total, 24 articles were included; 9 studies focused on the effect of high-fat meals compared with control; and 15 studies investigated the effects of different fat sources. We found that consumption of a high-fat meal causes a reduction in FMD (decrease in vasodilation) and AIx (decrease in arterial stiffness). For eicosapentaenoic acid/docosahexaenoic acid (from fish oil), postprandial assessment (FMD and AIx) indicates a beneficial effect on vascular function. There is limited evidence of an influence of CVD risk on the vascular response to meals with varying fat doses or FA composition. However, meaningful conclusions were difficult to draw because of the large heterogeneity of the studies. Inconsistent results regarding both the impact of fat dose and FA composition on postprandial vascular function should be noted. We propose standardized methods for postprandial protocols to improve data quality in future studies.

This review was registered in PROSPERO as CRD42022352986.

## Introduction

In the past few decades, the number of global, cardiovascular disease (CVD)-related deaths has steadily increased, from 12.1 million in 1990 to 18.6 million in 2019 [1]. According to the American Heart Association, an estimated 19.1 million people worldwide died in 2020 because of CVDs, with the highest age-standardized mortality rates in Eastern Europe and Central Asia [2]. Alongside increases in mortality, CVD-attributable disability-adjusted life years, years of life lost, and years lived with disability have increased considerably [1]. Atherosclerosis plays a critical role in the development of CVDs, including as an underlying cause of myocardial infarction and stroke [3]. During atherogenesis, an initially reversible fatty streak forms into a fibrous fatty lesion that becomes an atheroma [4]. Over time, this atherosclerotic plaque can rupture or erode, resulting in a cardiovascular event. The early stage of atherosclerosis is characterized by endothelial dysfunction [5,6]. Endothelial dysfunction describes a functional impairment of the endothelium. Characteristics include a decreased vasodilatation, a proinflammatory state, and prothrombotic properties [7]. Evidence also suggests an association between atherosclerosis and arterial stiffness [8,9]. Arterial stiffness is influenced by age and increased blood pressure [10], and structural alterations (e.g., fragmentation of elastic lamellae, increased collagen and calcium content) play a central role in its development [11].

In developed societies, humans spend ∼18 h of the day in a postprandial state [12] with continuously fluctuating diurnal lipemia [13]. Evidence suggests that postprandial lipemia leads to temporary, low-grade endothelial dysfunction mediated by local oxidative stress [[14], [15], [16], [17]]. According to current hypotheses, this enhanced oxidative stress reduces the availability of nitric oxide (NO) by increasing breakdown and reducing production [14]. NO plays an essential role in vasodilation, and reduced NO availability, resulting from either decreased production or activity, can cause endothelial dysfunction and contribute to atherosclerosis [18]. Thus, postprandial hypertriglyceridemia in response to a high-fat meal (HFM) may result in endothelial dysfunction, a marker of early stage of atherosclerosis, mediated by local oxidative stress and reduced NO availability.

There is convincing evidence that a dose–response relationship exists between total fat intake and postprandial triglyceride (TG) response [19]. In addition, 2 recent meta-analyses demonstrated that the fatty acid (FA) composition of a test meal influences the extent of postprandial lipemia [20,21]. Considering the detrimental effect of postprandial TGs on endothelial function, impaired vascular function is likely influenced by the amount and composition of fat ingested. Previous reviews on the acute effects of single HFMs on vascular function focused mainly on flow-mediated dilation (FMD) [22,23], revealing evidence of a marked decrease in FMD in the postprandial state compared with baseline values [23] and low-fat meals (LFMs) [22]. Concerning FA composition, evidence suggests an adverse effect of MUFAs [22] and a beneficial effect of long-chain n–3 (ω-3) PUFAs [24] on postprandial FMD. However, the overall evidence regarding the acute effects of FA composition on vascular function remains inconclusive [22,25].

In a recently published meta-analysis, Fewkes et al. [23] concluded that the effect size of an HFM on postprandial FMD is influenced by several factors, including age and BMI. Previously, we found that meals rich in SFAs provoke greater postprandial lipemia than meals with high amounts of unsaturated FA, especially in older subjects and/or subjects with elevated BMI [19]. These results suggest that especially in adults with certain CVD risk factors (e.g., obesity), high-fat doses and, in particular, SFA-rich meals, may have detrimental effects on postprandial vascular function.

Given this background, we aimed to systematically review and critically evaluate the existing evidence on the acute effects of fat dose and FA composition on vascular function assessed by FMD, pulse wave velocity (PWV), and augmentation index (AIx). An additional aim was to investigate whether acute changes in vascular function differ between metabolically healthy individuals and participants with increased CVD risk (e.g., those with obesity, metabolic syndrome, and hypertriglyceridemia). In addition, to maximize practical relevance, we focused on mixed meals.

## Methods

### Measurement of FMD, PWV, and AIx

Our analysis included noninvasive, yet reliable, measures of vascular function (FMD, PWV, and AIx) as outcome measures [[26], [27], [28], [29]]. Although FMD is the most well-established method to characterize endothelial function and reactivity [30], vessel stiffness is assessed by PWV and AIx [22]. FMD, PWV, and AIx are all independent predictors of cardiovascular events (e.g., fatal strokes) and all-cause mortality [[31], [32], [33]].

The FMD test was developed in 1992 by Celermajer et al. [34] and measures the endothelial-dependent vessel diameter change in response to blood flow-associated shear stress after a cuff occlusion period (recommended for 5 min) [26]. There are significant correlations between FMD and invasive measures of coronary artery changes [35] and brachial FMD and future cardiovascular events [36].

Likewise, PWV is an independent predictor of CVD risk and cardiovascular events [27]. PWV_c–f_ is calculated by dividing the distance between the common carotid artery and the common femoral artery by the transit time of the pulse wave between these points [28]. A higher PWV indicates a higher arterial stiffness [27]. In the 2018 European Society of Cardiology/European Society of Hypertension Guidelines for the management of arterial hypertension, the cut-off value for an influence of PWV_c–f_ on CVD risk was set at 10 m/s [37]. According to the European Network for Noninvasive Investigation of Large Arteries, PWV_c–f_ is regarded as the gold standard measurement of arterial stiffness [28].

Similar to the PWV, AIx serves as a surrogate parameter of arterial stiffness and these parameters correlate strongly [38]. The AIx is determined during pulse wave analysis [29]. It is a measure of wave reflection during systole and is usually adjusted to the heart rate by which it is influenced [27]. AIx correlates significantly with several CVD risk scores [29]. All 3 parameters of vascular function (FMD, PWV, and AIx) are reproducible [35,39].

### Literature search

**FIGURE 1:**
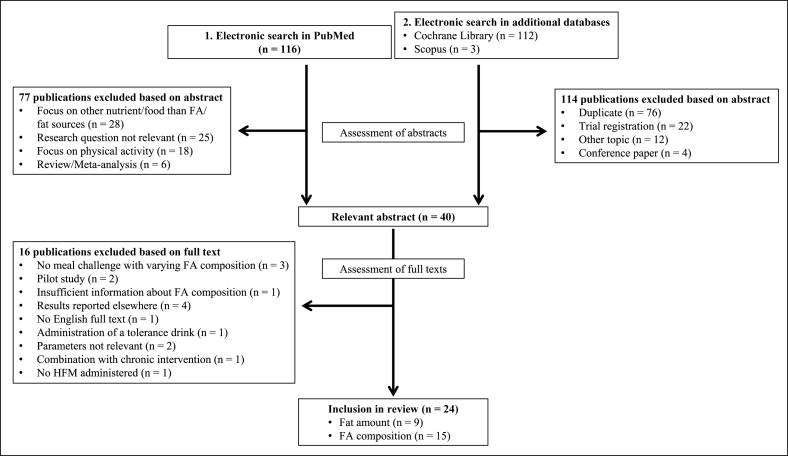
Flowchart of the article search and selection process. FA, fatty acid; HFM, high-fat meal.

To identify suitable studies, the databases of PubMed (https://pubmed.ncbi.nlm.nih.gov/), Scopus (https://www.scopus.com), and the Cochrane Library (https://www.cochranelibrary.com) were searched using the search term “postprandial AND fat AND meal AND (arterial stiffness OR flow mediated dilatation OR pulse wave velocity OR pulse wave analysis).” The initial database searches were conducted between July and August 2022, and the last update was made in June 2023. Both authors independently reviewed the identified papers and compared them with the inclusion and exclusion criteria. The main inclusion criteria (Table 1) were as follows: human intervention trial; adult participants; preparation of meals with fat sources (e.g., plant oils and dairy products); periodic measurement of postprandial FMD, PWV, and/or AIx; and paper written in English. To investigate the impact of HFMs on postprandial vascular function, studies were included if ≥1 HFM and 1 LFM were served or if ≥1 HFM meal was served and participants fasted as a control. To analyze the impact of the FA composition on postprandial vascular function, studies were included if the participants consumed ≥2 HFMs with varying FA compositions (e.g., SFA-rich compared with PUFA-rich). Articles were excluded if a tolerance test (e.g., fat tolerance test) was performed, or if chronic effects of total fat intake or FA composition were investigated. Furthermore, pilot studies and conference papers were excluded (Table 1). Studies were selected by consensus of both the authors (Figure 1). This review was registered in PROSPERO (CRD42022352986).

**TABLE 1:** Inclusion and exclusion criteria

Inclusion criteria	Exclusion criteria
1. Human intervention trial 2. Adult participants (≥18 y) 3. Preparation of meals with natural fat sources (e.g., plant oils) 4. At least 1 of the following comparisons was included: • HFM vs. LFM • HFM vs. fasting control • HFM vs. HFM with different FA compositions 5. Periodic measurement of postprandial FMD, PWV, and/or AIx 6. Paper in the English language	1. In vitro studies 2. Animal studies 3. Pilot studies 4. Conference papers 5. Chronic protocols 6. Tolerance tests (e.g., lipid or glucose tolerance tests)

Abbreviations: AIx, augmentation index; FA, fatty acid; FMD, flow-mediated dilation; HFM, high-fat meal; LFM, low-fat meal; PWV, pulse wave velocity.

## Results

Using the search string, 116 articles were identified during the systematic literature search in PubMed. On the basis of these abstracts, 77 publications were excluded because they did not fulfill the inclusion criteria and/or fulfilled ≥1 exclusion criterion (Table 1). The remaining 39 full texts were screened, of which 23 articles were rated as suitable for this review and thus included in the analysis. Using the same search string, literature searches in the Cochrane Library and Scopus were performed, revealing 112 and 3 publications, respectively. On the basis of the titles and abstracts, 76 duplicates were removed, and an additional 38 articles were excluded according to the inclusion and exclusion criteria (in total, 114 excluded abstracts). The remaining full text was assessed and rated as suitable for this review. In total, 24 publications were included in this analysis (Figure 1), which were published between 1999 and 2021.

### The impact of fat dose on postprandial vascular function

In the majority of studies that investigated the impact of fat dose on vascular function (TABLE 2, TABLE 3, TABLE 4), FMD (%) was the assessment measure used [[40], [41], [42], [43], [44], [45], [46], [47]]. Two studies provided data on AIx (%) [43,48], none measured PWV (m/s). In 7 trials, only healthy adults were investigated [40,[42], [43], [44], [45], [46], [47]], 1 study included subjects with coronary artery disease (CAD) [41], and 1 investigated lean adults, adults with obesity, and adults with type 2 diabetes mellitus (T2DM) [48]. Test meals were served as mixed meals consisting of commercially available foods (e.g., muffins, croissants, and milk) [40,41,[44], [45], [46],^48^] or as shakes [42,43,47] (TABLE 2, TABLE 3, TABLE 4). The amount of fat administered via test meals varied between studies from 0 to 18.4 g for the LFMs and from 50 to 95 g for the HFMs.

**TABLE 2:** Postprandial studies investigating the effects of fat dose on vascular function in healthy adults^1^

Reference	Study design	Age and BMI of subject group (*n*)	Meal components	Energy^2^ (kcal)	Macronutrient composition	FA composition	Parameter of vascular function (h)	Results
Bae et al. [40]	Parallel	HFM group 56 ± 6 y BMI N/A *n* = 11 (4 M, 7 F)	HFM 110 g rice 100 g Korean barbecue 20 g egg, 200 mL milk 8 g oil 25 g mayonnaise 50 g vegetable	HFM 803	HFM 53.4 g (59.9 E%) fat 30.7 g protein 50 g CHO	Both meals N/A	FMD (%) Ultrasound, brachial artery (0, 2 h)	HFM: significant decrease in FMD from baseline (*P* < 0.005) LFM: no significant postprandial change in FMD HFM, 2 h: significantly lower FMD compared with LFM (*P* = 0.037)
		LFM group 56 ± 12 y BMI N/A *n* = 9 (6 M, 3 F)	LFM 312 g rice 100 g vegetable soup 200 g vegetable 190 mL orange juice 400 g apple 50 g kimchi	LFM 802	LFM 3 g (3.4 E%) fat 15.7 g protein 178 g CHO			
Benson et al. [42]	Crossover	26 ± 3 y 24.7 ± 3.9 kg/m^2^*n* = 10 (10 M, 0 F)	HFM Milkshake (no further specification)	Both meals 11.6 kcal/kg BW	HFM 1 g/kg BW fat 0.15 g/kg protein 0.5 g/kg BW CHO	Both meals N/A	FMD (%, controlled for BMI) Ultrasound, brachial artery (0, 4 h)	HFM: significant decrease in FMD from baseline (*P* = 0.005) LFM: no significant postprandial change in FMD HFM: significantly greater FMD change compared with LFM (*P* < 0.05, meal x time interaction *P* = 0.046)
			LFM Low-fat, isoenergetic meal (no further specification)		LFM 0.04 g/kg BW fat 0.28 g/kg BW protein 2.54 g/kg BW CHO			
Esser et al. [43]	Crossover Randomized Double-blind	22 ± 2 y 22.7 ± 2.4 kg/m^2^*n* = 20 (20 M, 0 F)	HFM (500 mL) 53% fresh cream 3% sugar 44% water	HFM 954	HFM 95 g fat 6 g protein 22 g CHO	HFM 54 g SFA	FMD (%) Ultrasound, brachial artery (0, 3, 6 h)	Both meals, 3 h: significant decrease in FMD from baseline (*P* = 0.004); no significant group difference Both meals, 6 h: FMD returned to baseline
			LFM (500 mL) 43% full-cream milk 48% full-cream yogurt 4% lemonade 4% fantomalt 1% wheat fiber	LFM 400	LFM 14.5 g fat 17 g protein 49.5 g CHO	LFM 9 g SFA	AIx (%, corrected for heart rate) Applanation tonometry, radial artery (SphygmoCor) (0, 3, 6 h)	Both meals: significant decrease in AIx from baseline (*P* = 0.012); no significant group difference
Patik et al. [46]	Crossover Randomized	24 ± 3 y 24.3 ± 3.8 kg/m^2^*n* = 10 (10 M, 0 F)	HFM 1 egg muffin 1 sausage muffin 2 hash browns 591 mL water	HFM 990	HFM 55 g (50 E%) fat 35 g (14 E%) protein 89 g (36 E%) CHO	HFM 19 g SFA	FMD (%) Ultrasound, brachial artery (0, 2, 4 h)	HFM, 2 h: significant decrease from baseline compared with fasting condition (2 h: *P* = 0.002, 4 h: *P* = 0.004) and compared with HFM, 4 h *P* = 0.001) FMD change from baseline, 4 h: no significant difference between conditions
			Fasting state 591 mL water	Fasting state No energy intake	Fasting state No macronutrient intake	Fasting state No fat intake		
Poitras et al. [44]	Crossover^3^ Researcher analyzing images was blinded	23.2 ± 3.3 y 24.4 ± 2.4 kg/m^2^*n* = 10 (10 M, 0 F)	HFM 1 egg muffin 1 sausage muffin 2 hash browns Water	HFM 1000	HFM 54 g fat 32 g protein 94 g CHO	HFM 16 g SFA 1 g trans fat	FMD (%) Ultrasound, brachial artery (0, 1, 2, 3, 4 h)	No significant effect of meal or time on FMD
			LFM 160 g frosted flakes 500 mL skimmed milk 500 g orange juice	LFM 990	LFM 0 g fat 23 g protein 209 g CHO	LFM 0 g SFA 0 g trans fat		
Tushuizen et al. [45]	Crossover Randomized	25.4 ± 3 y 23.6 ± 1.8 kg/m^2^*n* = 17 (17 M, 0 F)	HFM: breakfast (0 h) 1 egg muffin 1 croissant with butter and marmalade 200 mL milk 20 mL cream HFM: lunch (4 h) 1 hamburger 1 croissant with butter 200 mL milk	HFM (each meal) 900	HFM (each meal) 50 g fat 30 g protein 55 g CHO	HFM (each meal) 60 % SFA (of total fat)	FMD (%) Ultrasound, brachial artery (0, 2, 4, 6, 8 h)	After lunch, 6 h: significant decrease in FMD from baseline (*P* < 0.05) Difference in FMD between interventions at 6 h: *P* = 0.051
			Fasting state Water (restricted to a maximum of 50 mL/h)	Fasting state No energy intake	Fasting state No macronutrient intake	Fasting state No fat intake		
Williams et al. [47]	Crossover^4^ Randomized	38 ± 6 y 24.6 ± 2.9 kg/m^2^*n* = 10 (10 M, 0 F)	HFM (shake) LFM enriched with 46 g unused cooking fat	HFM 897	HFM 64.4 g fat 20.5 g protein 62.5 g CHO	HFM 30 g SFA 4 g PUFA	FMD (%) Ultrasound, brachial artery (0, 4 h)	Both meals: no significant postprandial changes in FMD
			LFM (shake) 100 g ice cream 200 mL trim milk 50 mL evaporated milk 10 g yogurt 50 g tinned apricots (without syrup) 12 g egg yolk 30 g egg white Chocolate flavor	LFM 483	LFM 18.4 g fat 20.5 g protein 62.5 g CHO	LFM 8 g SFA 2 g PUFA		

Abbreviations: AIx, augmentation index; BW, body weight; CHO, carbohydrate; E%, energy percentage; FA, fatty acid; FMD, flow-mediated dilation; HFM, high-fat meal; LFM, low-fat meal; N/A, not available.

1 Age and BMI are given as mean ± SD.

2 Data on energy intake in MJ or kJ were converted to kcal (1 kcal = 4.184 kJ = 0.004184 MJ).

3 On 2 additional occasions, participants passed mental stress tasks; methods and results are not stated here.

4 A third test meal was enriched with used cooking fat; meal composition and results are not stated here.

**TABLE 3:** Postprandial studies investigating the effects of fat dose on vascular function in adults with CVD risk factors^1^

Reference	Study design	Age and BMI of subject group (*n*)	Meal components	Energy (kcal)	Macronutrient composition	FA composition	Parameter of vascular function (h)	Results
Bae et al. [41]	Parallel^2^	HFM group (CAD) 59 ± 11 y BMI N/A *n* = 9 (6 M, 3 F)	HFM 110 g rice 100 g Korean barbecue 20 g egg 200 mL milk 8 g oil 25 g mayonnaise 50 g vegetable	HFM 803	HFM 53.4 g (59.9 E%) fat 30.7 g protein 50 g CHO	Both meals N/A	FMD (%) Ultrasound, brachial artery (0, 2 h)	Both meals: no significant postprandial changes in FMD
		LFM group (CAD) 57 ± 11 y BMI N/A *n* = 9 (8 M, 1 F)	LFM 312 g rice 100 g vegetable soup 200 g vegetable 190 mL orange juice 400 g apple 50 g kimchi	LFM 802	LFM 3 g (3.4 E%) fat 15.7 g protein 178 g CHO			

Abbreviations: CHO, carbohydrate; E%, energy percentage; FA, fatty acid; FMD, flow-mediated dilation; HFM, high-fat meal; LFM, low-fat meal; N/A, not available.

1 Age and BMI are given as mean ± SD.

2 Two additional groups received angiotensin-converting enzyme inhibition and fibrates in addition to HFM; methods and results are not stated here.

**TABLE 4:** Postprandial studies investigating the effects of fat dose on vascular function in healthy adults and adults with CVD risk factors^1^

Reference	Study design	Age and BMI of subject group (*n*)	Meal components	Energy^2^ (kcal)	Macronutrient composition	FA composition	Parameter of vascular function (h)	Results
Phillips et al. [48]	Crossover Randomized	Lean adults 46.4 ± 10.7 y 23.2 (21.3, 24.2) kg/m^2^*n* = 8 (8 M, 0 F) Adults with obesity 40.9 ± 9.8 y 38.2 (31.8, 40.5) kg/m^2^*n* = 10 (10 M, 0 F) Adults with T2DM 56.3 ± 9.5 y 27.1 (26.5, 28.7) kg/m^2^*n* = 10 (10 M, 0 F)	HFM 1 bacon muffin 1 egg muffin 2 hash browns 1 caramel-flavored milk drink	HFM 989	HFM 57.5 g fat 35 g protein 83 g CHO	HFM 19.8 % SFA	AIx, AIx_75_ (%) Applanation tonometry, radial artery (SphygmoCor) (first h every 10 min, second h every 15 min, until 6 h every 30 min)	HFM, time course: decrease in AIx from baseline in all 3 groups (*P* value N/A) Fasting state, time course: no postprandial change in AIx in all 3 groups HFM, AIx iAUC: T2DM and lean subjects > obese subjects (obese vs. T2DM subjects *P* < 0.005; obese vs. lean subjects *P* < 0.05); difference across groups remained statistically significant when corrected for heart rate (75 bpm) HFM, AIx T2DM subjects: significant delay in time to return to baseline compared with lean subjects (*P* < 0.05)
			Fasting state Water (50 mL/h)	Fasting state No energy intake	Fasting state No macronutrient intake	Fasting state No fat intake		

Abbreviations: AIx, augmentation index; CHO, carbohydrate; FA, fatty acid; HFM, high-fat meal; iAUC, incremental AUC; N/A, not available; T2DM, type 2 diabetes mellitus.

1 Age and BMI are given as mean ± SD or median (interquartile range).

2 Data on energy intake in MJ or kJ were converted to kcal (1 kcal = 4.184 kJ = 0.004184 MJ).

### The effect of fat dose on FMD in healthy adults and those with CVD risk factors

In 7 studies, FMD (%) was measured in metabolically healthy adults [40,[42], [43], [44], [45], [46], [47]] (Table 2). In 5 of these, interventions consisted of an HFM and an LFM [40,[42], [43], [44],^47^]. Esser et al. [43] reported a small but significant decrease in FMD (%) from baseline 3 h after ingestion of the HFM (95 g fat) and LFM (14.5 g fat), without significant differences between the 2 meals. Bae et al. [40] and Benson et al. [42] observed a significant decrease in FMD (%) from preprandial values in response to the HFM (fat content: 53.4 g, 1 g/kg body weight [BW]), but did not detect a significant change in FMD (%) after consumption of the LFM (fat content: 3 g, 0.04 g/kg BW). In contrast, Poitras et al. [44] and Williams et al. [47] found no effect of either an HFM (fat content: 54 g, 64.4 g) or LFM (fat content: 0 g, 18.4 g) on postprandial FMD (%) (Table 2). In 2 studies [45,46], FMD (%) was measured after consumption of 1 or 2 consecutive HFMs and on a different day during a fasting period (Table 2). Tushuizen et al. [45] reported a significant decrease in FMD (%) from baseline after a fat-rich lunch that was ingested 4 h after a fat-rich breakfast (each meal: 50 g fat). The difference in FMD (%) at the equivalent time point during the fasting protocol tended toward statistical significance (*P* = 0.051). Patik et al. [46] observed that 2 h postprandially, a fat-rich, fast-food meal (55 g fat) led to a significant decrease in FMD (%) from baseline compared with the fasting protocol; however, 2 h later, there was no significant difference in FMD (%) change from baseline between conditions. In both studies, the fasting state had no influence on FMD (%) (Table 2).

The literature search revealed one study in which the effects of an HFM (53.4 g fat) and an LFM (3 g fat) on FMD (%) were measured in participants with enhanced CVD risk (Table 3). Bae et al. [41] reported no significant effects of the test meals on postprandial FMD (%) in adults with CAD.

### The effect of fat dose on AIx and PWV in healthy adults and those with CVD risk factors

Two studies investigated the impact of an HFM on AIx (%) [43,48] (TABLE 2, TABLE 4). In healthy adults, Esser et al. [43] observed a significant decrease in heart rate corrected AIx (%) from baseline after eating both an HFM (95 g fat) and an LFM (14.5 g fat), with no significant difference between groups (Table 2). Phillips et al. [48] reported that in lean adults, nondiabetic adults with obesity, and individuals with T2DM, AIx (%) decreased from baseline after consumption of an HFM (57.5 g fat) but remained unchanged during the fasting period (Table 4).

No studies investigating the effects of fat dose on vascular function measured PWV (m/s).

### The impact of FA composition on postprandial vascular function

In total, 15 studies were included that analyzed the impact of FA composition on vascular function (Tables 5–**7**). Seven were performed in healthy adults [[49], [50], [51], [52], [53], [54], [55]] and 6 in adults with CVD risk factors [[56], [57], [58], [59], [60], [61]]. The remaining 2 studies [62,63] included both healthy adults and adults with increased CVD risk. Berry et al. [49] measured all 3 parameters of vascular function (FMD [%], PWV [m/s], and AIx [%]), Lithander et al. [50] and McManus et al. [57] determined AIx (%) and PWV (m/s), and Kendall et al. [56] and Chong et al. [55] only measured AIx (%). Most studies supplied data on FMD (%) but not AIx (%) or PWV (m/s) [[51], [52], [53], [54],[58], [59], [60], [61], [62], [63]]. In many papers, the meals consisted of a shake or drink (e.g., a chocolate drink), either entirely [50,60] or partially [49,51,55,[57], [58], [59]]. The test meals were enriched with a pure fat source (e.g., olive oil [50,[61], [62], [63]]), an oil blend (e.g., palm/soy bean oil mixture [55,57]), or they contained fat-rich foods (e.g., burger and French fries [52], various dairy products [58]) (TABLE 5, TABLE 6, TABLE 7). The fat amount consumed via the test meals ranged from 29 to 80 g.

**TABLE 5:** Postprandial studies investigating the effects of FA composition on vascular function in healthy adults^1^

Reference	Study design	Age and BMI of subject group (*n*)	Meal components	Energy^2^ (kcal)	Macronutrient composition	Fat source(s)	FA composition	Parameter of vascular function (h)	Results
Berry et al. [49]	Crossover Randomized	27.1 ± 5.3 y 24.3 ± 3.0 kg/m^2^*n* = 17 (17 M, 0 F)	Both meals 2 muffins (each containing 25 g test fat) 1 milkshake	Both meals 853	Both meals 50 g fat 15 g protein 89 g CHO	Meal rich in stearic acid Shea butter blend (refined shea butter blended with small amount of sunflower oil)	Meal rich in stearic acid 26.7 g C18:0 16.6 g C18:1 n–9 4.5 g C18:2 n–6	FMD (%) Ultrasound, brachial artery (0, 3 h)	Significant meal x time interaction for FMD (*P* = 0.039) High-oleic sunflower oil: significant decrease from baseline (*P* < 0.001) Shea butter blend: no significant postprandial change in FMD Change in FMD, 3 h: significant difference between meals (*P* < 0.05)
						Meal rich in oleic acid High-oleic sunflower oil	Meal rich in oleic acid 0.8 g C18:0 42.5 g C18:1 n–9 4.0 g C18:2 n–6	PWV_c–f_ (m/s) Applanation tonometry, carotid and femoral artery (SphygmoCor) (0, 3 h)	Both meals: no significant postprandial changes in PWV_c–f_
								AIx (%) Applanation tonometry, radial artery (SphygmoCor) (0, 3 h)	Significant time effect (decrease after both meals) for central AIx (*P* = 0.019) and peripheral AIx (*P* < 0.001) Changes in central AIx and peripheral AIx, 3 h: no significant differences between meals
Chong et al. [55]	Crossover Randomized Single-blind	48 ± 18 y 24.7 ± 3.2 kg/m^2^*n* = 25 (12 M, 13 F)	Both meals^3^ Chocolate milkshake (containing 30 g test fat) 3 slices white bread Strawberry jam	Both meals N/A	Both meals 33.3 g fat 23 g protein 138 g CHO	Control meal Palm olein and soybean oil (4:1)	Both meals N/A	AIx_75_ (%) Applanation tonometry, radial artery (SphygmoCor) (0, 0.5, 1, 1.5, 2, 3, 4 h)	Significant treatment and time effects (both: *P* = 0.02) LC n–3 PUFA: attenuating effect on AIx_75_ compared with control (more moderate increase toward baseline subsequent to AIx_75_ reduction)
						LC n–3 PUFA-rich meal 23.2 g control oil, 6.8 g fish oil (2.0 g EPA, 2.7 g DHA)			
Lithander et al. [50]	Crossover Randomized Single-blind	38.7 ± 14.4 y 24.1 ± 2.3 kg/m^2^*n* = 20 (20 M, 0 F)	SFA-rich meal (shake) Whole milk Skimmed milk powder Instant drink powder (strawberry flavor) Water Double cream Sunflower oil 400 ml water	SFA-rich meal 747	SFA-rich meal 57.6 g fat 18.8 g protein 41.1 g CHO	SFA-rich meal Double cream Sunflower oil	SFA-rich meal 33.84 g SFA (5.31 g C14:0 14.29 g C16:0 5.9 g C18:0) 14.64 g MUFA 4.18 g PUFA	AIx, AIx_75_ (%) Applanation tonometry, radial artery (SphygmoCor) (0, 0.5, 1, 1.5, 2, 2.5, 3, 3.5, 4 h)	Significant decrease in AIx and AIx_75_ from baseline (time effect for both *P* < 0.01); AIx no longer significant after adjustment for heart rate and MAP increase; AIx_75_ significant after adjustment for MAP increase (*P* < 0.05) No significant differential effects of meal type
			MUFA-rich meal (shake) Whole milk Skimmed milk powder Instant drink powder (strawberry flavor) Water Olive oil 400 ml water	MUFA-rich meal 712	MUFA-rich meal 54.5 g fat 17.4 g protein 39.7 g CHO	MUFA-rich meal Olive oil	MUFA-rich meal 11.7 g SFA 36.42 g MUFA (35.4 g C18:1 n–9) 3.97 g PUFA	PWV_c–f_ (m/s) Applanation tonometry, carotid and femoral artery (SphygmoCor) (0, 0.5, 1, 1.5, 2, 2.5, 3, 3.5, 4 h)	Significant increase in PWV_c–f_ from baseline (time effect *P* < 0.05); no longer significant after adjustment for MAP increase No significant differential effects of meal type
Nicholls et al. [51]	Crossover Randomized Single-blind (investigator)	29.5 ± 2.3 y 23.6 ± 0.8 kg/m^2^*n* = 14 (8 M, 6 F)	Both meals Slice of carrot cake Milkshake	Both meals N/A	Both meals 1 g/kg BW fat	PUFA-rich meal Safflower oil	PUFA-rich meal 8.8 % SFA 13.6 % MUFA 75 % PUFA	FMD (%) Ultrasound, brachial artery (0, 3, 6 h)	FMD, 3 h: significant decrease from baseline following coconut oil (*P* < 0.05); no significant postprandial change following safflower oil Change in FMD: no significant group difference FMD, 6 h, both meals: no significant change in FMD from fasting values
						SFA-rich meal Coconut oil	SFA-rich meal 89.6 % SFA 5.8 % MUFA 1.9 % PUFA		
Raitakiri et al. [53]	N/A	33 ± 7 y 24.3 ± 3.1 kg/m^2^ Meal 1 *n* = 12 (7 M, 5 F) Meal 2 *n* = 10 (restudied, sex N/A)	Meal 1, SFA-rich 1 sausage 2 muffins 2 hash browns (cooked in 61 g fresh tallow)	SFA-rich meal 1030	SFA-rich meal N/A	SFA-rich meal Fresh tallow	SFA-rich meal 48 % SFA 40 % MUFA 7.4 % PUFA 4.6 % trans FA	FMD (%) Ultrasound, brachial artery (0, 3, 6 h)	Both meals: no significant postprandial changes in FMD
			Meal 2, MUFA-rich Similar constituents, fat content and energy amount (not further specified) Different FA composition				MUFA-rich meal 10 % SFA 85 % MUFA 5 % PUFA		
Rudolph et al. [52]	Crossover Randomized Single-blind (observer)	32 ± 11 y 24 ± 5 kg/m^2^*n* = 24 (10 M, 14 F)	Beef burger meal 211 g beef burger 152 g French fries 20 mL Ketchup 500 mL soft drink	Beef burger meal 1245	Beef burger meal 49 g fat 33 g protein 158 g CHO	Beef burger meal Burger French fries	Beef burger meal 13.1 g SFA 7.8 g trans FA	FMD (%) Ultrasound, brachial artery (0, 2, 4 h)	All meals: significant decrease in FMD from baseline over time (time effect *P* < 0.001); no significant differences between meals (no significant meal type effect)
			Vegetarian burger meal 1 203 g vegetarian burger 152 g French fries 20 mL ketchup 500 mL soft drink	Vegetarian burger meal 1 1216	Vegetarian burger meal 1 49 g fat 17 g protein 167 g CHO	Vegetarian burger meal 1 Burger French fries	Vegetarian burger meal 1 5.0 g SFA 6.9 g trans FA		
			Vegetarian burger meal 2 203 g vegetarian burger 90 g salad 30 mL dressing 306 g yogurt (fruit) 500 mL orange juice	Vegetarian burger meal 2 1057	Vegetarian burger meal 2 31 g fat 25 g protein 161 g CHO	Vegetarian burger meal 2 Burger Dressing Yogurt	Vegetarian burger meal 2 4.0 g SFA 0.3 g trans FA		
Volpe et al. [54]	Crossover^4^ Randomized Single-blind (observer)	40 ± 11 y 26 ± 4 kg/m^2^*n* = 18 (18 M, 0 F)	SFA-rich meal 3 ounces of bacon 1 slice of processed cheese 2 servings of egg substitute 5 large black olives 1 bagel 1.3 tbsp yogurt-based margarine 0.75 cup canned pears in water 8 oz milk (1% fat)	Both meals 700	SFA-rich meal 29 g fat 43 g protein 65 g CHO	SFA-rich meal Various foods (SFA-rich)	SFA-rich meal 10 g SFA	FMD (%) Ultrasound, brachial artery (0, 3 h)	Both meals: no significant postprandial changes in FMD
			PUFA-rich meal 5 oz salmon 1 bagel 2.25 tbsp yogurt-based margarine 1 tbsp cashew butter 0.5 tbsp parmesan cheese 1.5 tbsp walnuts 0.25 cup canned peaches in water 8 oz mineral water		PUFA-rich meal 29 g total fat 44 g protein 65 g CHO	PUFA-rich meal Various foods (PUFA-rich)	PUFA-rich meal 5 g SFA 4 g n–3-FA		

Abbreviations: AIx, augmentation index; BW, body weight; CHO, carbohydrate; E%, energy percentage; FA, fatty acid; FMD, flow-mediated dilation; LC, long chain; MPA; N/A, not available; PWV, pulse wave velocity.

1 Age and BMI are given as mean ± SD.

2 Data on energy intake in MJ or kJ were converted to kcal (1 kcal = 4.184 kJ = 0.004184 MJ).

3 Test meals were consumed 5 h after a low-fat, standard breakfast (400 kcal, 2.1 g of fat).

4 In addition to healthy adults, HIV-infected adults with and without antiretroviral therapy were studied; methods and results are not stated here.

**TABLE 6:** Postprandial studies investigating the effects of FA composition on vascular function in adults with CVD risk factors^1^

Reference	Study design	Age and BMI of subject group (*n*)	Meal components	Energy^2^ (kcal)	Macronutrient composition	Fat source(s)	FA composition	Parameter of vascular function (h)	Results
Kendall et al. [56]	Crossover^3^ Randomized	54 ± 8 y 37.5 ± 7.9 kg/m^2^*n* = 20 (8 M, 12 F) Metabolic syndrome	SFA-rich meal 110 g white bread 19 g butter 80 g cheese	SFA-rich meal 704.3	SFA-rich meal 42.3 g fat 29.3 g protein 50.0 g available CHO	SFA-rich meal Butter Cheese	SFA-rich meal 26.8 g SFA 11.7 g MUFA 1.6 g PUFA	AIx (%) Pulse amplitude tonometry (Endo-PAT) (0, 1, 3 h)	Both meals: significant decrease in AIx from baseline (*P* value N/A); no significant group differences
			MUFA-/PUFA-rich meal 85 g white bread 85 g pistachios	MUFA-/PUFA-rich meal 705.4	MUFA-/PUFA-rich meal 41.9 g fat 29.1 g protein 50.1 g available CHO	MUFA-/PUFA-rich meal Pistachios	MUFA-/PUFA-rich meal 5.1 g SFA 21.9 g MUFA 12.7 g PUFA		
Markey et al. [58]	Crossover Randomized Double-blind	53 ± 2 y 25.9 ± 0.5 kg/m^2^*n* = 52 (31 M, 21 F) Moderate CVD risk	Breakfast 75 g white bread 32.6 g cheddar cheese 29.4 g butter (control meal) 32.6 g butter (modified meal) 38 g cornflakes 195 g milk Milkshake (330 g milk, 19 g strawberry sauce)	Control breakfast 980	Control breakfast 49.9 g fat 39.7 g protein 101.4 g CHO	Control meals Various conventional dairy products	Control breakfast 31.7 g SFA 12.3 g MUFA 2.8 g PUFA 2.2 g trans FA	FMD (%) Ultrasound, brachial artery (0, 3, 5, 7 h)	No significant effect on FMD (time-course profile, overall treatment) or difference in FMD between meal types (AUC, iAUC)
				Control lunch 598	Control lunch 30.3 g fat 19.6 g protein 63.3 g CHO		Control lunch 19.1 g SFA 7.4 g MUFA 1.8 g PUFA 1.4 g trans FA		
			Lunch 60 g white bread 15 g cheddar cheese 18.6 g butter (control meal) 19.8 g butter (modified meal) Milkshake (control: 350 g milk, modified: 352 g milk, both: 27 g strawberry sauce)	Modified breakfast 1028	Modified breakfast 50.6 g fat 36.1 g protein 105.9 g CHO	Modified meals Various dairy products with modified FA composition^4^	Modified breakfast 24.5 g SFA 20.0 g MUFA 2.9 g PUFA 3.9 g trans FA		
				Modified lunch 621	Modified lunch 30.6 g fat 20.9 g protein 64.6 g CHO		Modified lunch 14.8 g SFA 12.1 g MUFA 1.8 g PUFA 2.6 g trans FA		
McManus et al. [57]	Crossover Randomized Double-blind	45 ± 5 y 27.4 ± 3.3 kg/m^2^*n* = 26 (26 M, 0 F) Increased CVD risk	All test meals Milk shake (40 g test fat 150 g skimmed milk 15 chocolate-flavored powder 15 skimmed milk powder 2 g peppermint oil extract) 73 g white bread 30 g jam	All test meals 748	All test meals 51.0 E% (42.4 g) fat 9.5 E% protein 39.5 E% CHO	Control meal 4:1 palm oil and soybean oil mixture	Control meal N/A	AIx (%) Oscillometric device, brachial artery (Vicorder) (0, 4 h)	Significant time effect (*P* < 0.010) and time x treatment interaction (*P* = 0.005) Post hoc analysis: significantly greater reduction in AIx after DHA-containing meal compared with control meal (*P* = 0.047); comparison of EPA-containing meal and control meal reached borderline significance (*P* = 0.06)
						EPA-containing meal 6.94 g of oil mixture replaced by EPA-rich oil	EPA-containing meal 4.16 g EPA (not further specified)	PWV_c–f_ (m/s) Oscillometric device, carotid and femoral artery (Vicorder) (0, 4 h)	All meals: no significant postprandial changes in PWV
						DHA-containing meal 8.33 g of oil mixture replaced by DHA-rich oil	DHA-containing meal 4.16 g DHA (not further specified)		
Rathnayake et al. [59]	Crossover Randomized Double-blind	58 ± 1 y 25.9 ± 0.7 kg/m^2^*n* = 32 (0 M, 32 F) Postmenopausal women	SFA breakfast Chocolate drink (containing 42 g test fat) Toast with strawberry jam and test fat (20 g)	SFA breakfast 908	SFA breakfast 53.7 g fat 19.6 g protein 98.4 g CHO	SFA breakfast Butter (62 g)	SFA breakfast 32.9 g SFA 13.3 g MUFA 1.8 g n–6 PUFA 0.6 g n–3 PUFA 1.95 g trans FA	FMD (%) Ultrasound, brachial artery (0, 3, 5, 7 h)	No significant effect on FMD (time-course profile, overall treatment) or difference in FMD between meal types (AUC, iAUC)
			SFA lunch Chocolate drink (containing 15 g test fat) Toast with strawberry jam and test fat (20 g)	SFA lunch 717	SFA lunch 31.8 g fat 19.5 g protein 98.2 g CHO	SFA lunch Butter (35 g)	SFA lunch 19.1 g SFA 7.7 g MUFA 1.3 g n–6 PUFA 0.3 g n–3 PUFA 1.12 g trans FA		
			MUFA breakfast Chocolate drink (containing 36 g test fat) Toast with strawberry jam and test fat (17 g)	MUFA breakfast 908	MUFA breakfast 53.1 g fat 19.2 g protein 98.0 g CHO	MUFA breakfast Refined olive oil (17 g) Olive oil and rapeseed oil-blended spread (36 g)	MUFA breakfast 9.4 g SFA 35.2 g MUFA 5.1 g n–6 PUFA 0.9 g n–3 PUFA 0.13 g trans FA		
			MUFA lunch Chocolate drink (containing 15 g test fat) Toast with strawberry jam and test fat (15 g)	MUFA lunch 717	MUFA lunch 31.1 g fat 19.2 g protein 98.0 g CHO	MUFA lunch Refined olive oil (15 g) Olive oil and rapeseed oil-blended spread (15 g)	MUFA lunch 6.1 g SFA 19.4 g MUFA 3.4 g n–6 PUFA 0.6 g n–3 PUFA 0.12 g trans FA		
			n–6 PUFA breakfast Chocolate drink (containing 36 g test fat) Toast with strawberry jam and test fat (17 g)	n–6 PUFA breakfast 908	n–6 PUFA breakfast 53.1 g fat 19.2 g protein 98.0 g CHO	n–6 PUFA breakfast Safflower oil (17 g) Safflower oil spread (36 g)	n–6 PUFA breakfast 7.6 g SFA 6.7 g MUFA 36.2 g n–6 PUFA 0.1 g n–3 PUFA 0.12 g trans FA		
			n–6 PUFA lunch Chocolate drink (containing 14 g test fat) Toast with strawberry jam and test fat (17 g)	n–6 PUFA lunch 717	n–6 PUFA lunch 31.1 g fat 19.2 g protein 98.0 g CHO	n–6 PUFA breakfast Safflower oil (17 g) Safflower oil spread (14 g)	n–6 PUFA breakfast 5.4 g SFA 4.1 g MUFA 20.0 g n–6 PUFA 0.1 g n–3 PUFA 0.12 g trans FA		
Stonehouse et al. [61]	Crossover Randomized Double-blind	56.8 (53.7, 59.8) y 30.0 (28.7, 31.3) kg/m^2^*n* = 28 (28 M, 0 F) Overweight and obese	Both meals 200 g chicken (fried in 40 g of test oil) Fried white bread Small salad (20 g lettuce, 10 g tomato, 10 g cucumber)	Both meals 667	Both meals 44 g (58 E%) fat 40 g (30 E%) protein 21 g (11 E%) CHO	SFA-rich meal Refined bleached deodorized palm olein	SFA-rich meal 41.9 % SFA (36.2 % C16:0) 46.8 % MUFA (46.1 % C18:1 n–9) 11.5 % PUFA (11.3 % C18:2 n–6)	FMD (%) Ultrasound, brachial artery (0, 1, 2, 3, 4, 5 h)	Both meals: no significant postprandial changes in FMD No difference in FMD response between meals
						MUFA- rich meal Olive oil	MUFA- rich meal 16.6% SFA (11.7% C16:0) 76.2% MUFA (74.1% C18:1 n–9) 7.25% PUFA (6.8% C18:2 n–6)		
West et al. [60]	Crossover Randomized Double-blind	T2DM, low TGs 51.4 ± 2.3 y 29.6 ± 1.4 kg/m^2^*n* = 10 (80 % M, 20 % F) T2DM, high TGs 59.6 ± 3.2 y 28.6 ± 0.8 kg/m^2^*n* = 8 (62 % M, 38 % F)	All test meals 473 mL skimmed milk 50 g test oil Ice Flavorings	All test meals 625	All test meals 50 g (72 E%) fat 20 g (13 E%) protein 24 g (15 E%) CHO	MUFA meal High-oleic safflower oil (90%) Canola oil (10%)	MUFA meal 4.5 g SFA 32.6 g MUFA 9.8 g PUFA (9.2 g C18:2 n–6 0.5 g C18:3 n–3)	FMD (%) Ultrasound, brachial artery (0, 4 h)	T2DM, low TGs All meals: no significant postprandial changes in FMD T2DM, high TGs MUFA meal: no significant postprandial change in FMD MUFA + ALA meal and MUFA + EPA/DHA meals: significant increases in FMD from baseline (*P* ≤ 0.04) FMD change: significant treatment x group interaction (*P* < 0.03)
						MUFA + ALA meal Canola oil (70%) High-oleic safflower oil (20%) Safflower oil (10%)	MUFA + ALA meal 3.5 g SFA 31.2 g MUFA 12.8 g PUFA (9.2 g C18:2 n–6 3.3 g C18:3 n–3)		
						MUFA + EPA/DHA meal High-oleic safflower oil (60%) Safflower oil (25%) Sardine oil (15%)	MUFA + EPA/DHA meal 5.0 g SFA 30.7 g MUFA 11.8 g PUFA (6.1 g C18:2 n–6 4.8 g n–3 FA 0.2 g C18:3 n–3 2.76 g C20:5 n–3 1.16 g C22:6 n–3)		

Abbreviations: AIx, augmentation index; CHO, carbohydrate; CVD, cardiovascular disease; E%, energy percentage; FA, fatty acid; FMD, flow-mediated dilation; iAUC, incremental AUC; N/A, not available; PWV, pulse wave velocity; T2DM, type 2 diabetes mellitus.

1 Age and BMI are given as mean ± SD or mean (95 % CI).

2 Data on energy intake in MJ or kJ were converted to kcal (1 kcal = 4.184 kJ = 0.004184 MJ).

3 Three additional test meals consisted of white bread (12 and 50 g available CHO) and pistachios; the nutrient composition and results of these meals are not stated here.

4 SFA content was decreased and MUFA content was increased by a “high-oleic sunflower oil dairy-cow feeding strategy.”

**TABLE 7:** Postprandial studies investigating the effects of FA composition on vascular function in healthy adults and adults with CVD risk factors^1^

Reference	Study design	Age and BMI of subject group (*n*)	Meal components	Energy (kcal)	Macronutrient composition	Fat source(s)	FA composition	Parameter of vascular function (h)	Results
Cortés et al. [62]	Crossover Randomized	Healthy control 32 ± 8 y 24.7 ± 3.0 kg/m^2^*n* = 12 (9 M, 3 F) Hypercholesterolemic 45 ± 13 y 26.3 ± 3.5 kg/m^2^*n* = 12 (11 M, 1 F)	Both meals 100 g white bread 75 g salami 50 g fatty cheese 125 yogurt (10%) Water ad libitum In addition Olive oil or walnuts	Both meals 1200	Both meals 63% fat (80 g total fat) 15% protein 22% CHO	Olive oil meal 25 mL olive oil	Olive oil meal 35% SFA 38 % MUFA (olive oil: 78% C18:1 n–9) 7% PUFA	FMD (%) Ultrasound, brachial artery (0, 4 h)	Healthy control Olive oil: decrease in FMD from baseline (−17%) Walnut meal: no postprandial change in FMD Hypercholesterolemic Olive oil: decrease in FMD from baseline (−36%) Walnut meal: increase in FMD from baseline (+24%)
						Walnut meal 40 g shelled walnuts	Walnut meal 35% SFA 23% MUFA 23% PUFA (5.4 g C18:3 n–3)		
Cutruzzolà et al. [63]	Crossover Randomized Sonographer was blinded to meal type	Healthy control 25 ± 3 y 22.3 ± 1.8 kg/m^2^*n* = 6 (6 M, 0 F) T1DM 28 ± 8 y 24.2 ± 3.0 kg/m^2^*n* = 10 (7 M, 3 F)	Both meals 80 g white rice 200 g potatoes 140 g lean beef 100 mL water In addition Butter or extra virgin olive oil	Both meals 900	Butter meal 39.7 g (40%) fat 40.9 g (17%) protein 100.5 g (43%) CHO	Butter meal 40 g butter	Butter meal 21.7 g SFA 11.6 g MUFA 2.6 g PUFA	FMD (%) Ultrasound, brachial artery (0, 1, 3, 5 h)	Healthy control No significant postprandial changes in FMD T1DM Significantly higher FMD after olive oil meal compared with butter meal (*P* = 0.007)
					Olive oil meal 41.1 g (40%) fat 38.5 g (17%) protein 100.1 g (43%) CHO	Olive oil meal 35 g extra virgin olive oil	Olive oil meal 7.7 g SFA 28.0 g MUFA 4.6 g PUFA		

Abbreviations: CHO, carbohydrate; CVD, cardiovascular disease; FA, fatty acid; FMD, flow-mediated dilation; T1DM, type 1 diabetes mellitus.

1 Age and BMI are given as mean ± SD.

### The effect of FA composition on FMD in healthy adults and those with CVD risk factors

The literature search revealed 7 studies investigating the impact of FA composition on FMD (%) in healthy adults [49,[51], [52], [53], [54],^62^,^63^] (TABLE 5, TABLE 7). Of these, 3 found that a HFM led to a decrease in FMD (%) from baseline, while another meal, enriched with a different vegetable fat source, had no influence on postprandial FMD (%) [49,51,62]. Specifically, Berry et al. [49] reported a decrease in FMD (%) from baseline after a meal enriched with high-oleic sunflower oil (Table 5), whereas Cortés et al. [62] reported similar results after a meal enriched with olive oil (also rich in oleic acid) (Table 7). By contrast, meals enriched with a shea butter blend (refined shea butter blended with sunflower oil, rich in stearic acid) or shelled walnuts (rich in linoleic acid) had no influence on postprandial FMD (%) [49,62]. Nicholls et al. [51] reported a significant decrease in FMD (%) from baseline 3 h after consumption of a SFA-rich meal (enriched with coconut oil) but not in response to a PUFA-rich meal (enriched with safflower oil) (Table 5); responses were not significantly different between meals. After 6 h, the effect of the SFA-rich meal on FMD (%) was no longer significantly different compared with the baseline. Rudolph et al. [52] observed that 3 fast-food meals administered with varying FA profiles (providing different amounts of SFAs and trans FAs) provoked significant reductions in FMD (%) from baseline without significant differences between meals (Table 5). The remaining 3 studies did not detect significant postprandial changes in FMD (%) subsequent to test meals [53,54,63] (TABLE 5, TABLE 7). Meals contained SFA- and MUFA-rich foods [53], SFA- and PUFA-rich foods [54], or butter (SFA-rich) and olive oil (MUFA-rich) as fat sources [63].

In addition to healthy adults, 2 of the above-mentioned studies also included hypercholesterolemic individuals [62] and subjects with type 1 diabetes mellitus (T1DM) [63] (Table 7). In hypercholesterolemic individuals, Cortés et al. [62] reported a decrease in FMD (%) from baseline in response to a meal enriched with olive oil (rich in oleic acid), whereas FMD (%) increased from baseline after ingestion of the meal with shelled walnuts (rich in linoleic acid). Cutruzzolà et al. [63] found that compared with a meal enriched with butter (rich in SFAs, especially lauric acid), FMD (%) was significantly higher after a meal containing extra virgin olive oil (predominantly composed of MUFAs) (Table 7). Four further studies, including adults with CVD risk factors were analyzed [[58], [59], [60], [61]] (Table 6). Three did not detect significant differences in FMD (%) after ingestion of meals enriched with refined bleached deodorized palm olein or olive oil [61], a breakfast and lunch containing conventional dairy products or FA-modified dairy products (decreased SFA amount and increased MUFA amount) [58], or meals enriched with fat sources consisting mainly of SFAs (butter), MUFAs (refined olive oil and olive oil and canola oil-blended spread), or n–6 PUFAs (safflower oil and spread) [59] (Table 6). West et al. [60] included adults with T2DM and differentiated between individuals with high- and low fasting TGs. In the group with low fasting TGs, FMD (%) did not change significantly in response to test meals rich in MUFAs (fat source: high-oleic safflower oil, canola oil), MUFAs + α-linolenic acid (ALA) (fat sources: canola oil, high-oleic safflower oil, and safflower oil), or MUFAs + EPA/DHA (fat sources: high-oleic safflower oil, safflower oil, and sardine oil). In the group with high fasting TGs, there was no change in FMD (%) following the MUFA meal, but FMD (%) showed a significant increase from baseline 4 h after ingestion of the MUFA + ALA and the MUFA + EPA/DHA meal, leading to a significant treatment–group interaction (Table 6).

### The effect of FA composition on AIx in healthy adults and those with CVD risk factors

The literature search revealed 3 studies that investigated the effects of FA composition on AIx (%) in healthy subjects [49,50,55] (Table 5). Berry et al. [49] reported a significant decrease in central and peripheral AIx (%) from baseline in response to test meals enriched with shea butter blend (refined shea butter blended with sunflower oil, rich in stearic acid) and high-oleic sunflower oil (Table 5); there was no significant difference between test meals. Similarly, Lithander et al. [50] observed a significant reduction in AIx (%) and AIx_75_ (%, standardized to a heart rate of 75 bpm) from baseline after a SFA-rich meal (fat sources: double cream, sunflower oil; rich in palmitic acid) and a MUFA-rich meal (fat source: olive oil; rich in oleic acid) (Table 5). The effect on AIx_75_ (%) remained significant after adjustment for mean arterial pressure (MAP), whereas the effect on AIx (%) was no longer significant when adjusted for increases in heart rate and MAP. There was no significantly different effect of MUFA-rich meal compared with SFA-rich meal on AIx or AIx_75_ (%). Chong et al. [55] found that following a postprandial AIx_75_ (%) reduction in response to both meals, the AIx_75_ (%) increased to a lower extent after a meal enriched with EPA and DHA compared with a control meal (fat source: palm olein and soybean oil) (Table 5).

Two publications investigated the effects of FA composition on AIx (%) in adults with CVD risk factors [56,57] (Table 6). Kendall et al. [56] reported that in response to white bread, butter, and cheese (high SFA content), as well as to white bread and pistachios (high MUFA and PUFA content), AIx (%) decreased from baseline in subjects with metabolic syndrome (Table 6). However, the change from fasting was not significantly different between meals. McManus et al. [57] observed that compared with a meal enriched with palm and soy bean oil (control meal), the decrease in AIx (%) was significantly greater when subjects with CVD risk factors consumed a meal enriched with palm oil, soy bean oil, and DHA (palm and soybean oil mixture partly replaced by DHA-rich oil). Compared with the control meal, a meal enriched with palm oil, soy bean oil, and EPA (palm and soybean oil mixture partly replaced by EPA-rich oil) tended to cause a greater decrease in AIx (%, *P* = 0.06) (Table 6).

### The effect of FA composition on PWV in healthy adults and those with CVD risk factors

In 2 of the above-mentioned studies, PWV (m/s) was measured to analyze the effect of FA composition on arterial stiffness in healthy subjects [49,50] (Table 5). Compared with baseline values, Berry et al. [49] reported no changes in PWV_c–f_ (m/s) measured 3 h after ingestion of meals enriched with high-oleic sunflower oil or shea butter blend (refined shea butter blended with sunflower oil, rich in stearic acid) (Table 5). Regardless of the FA composition of test meals (SFA meal rich in palmitic acid compared with MUFA meal rich in oleic acid), Lithander et al. [50] observed a significant increase in PWV_c–f_ (m/s) from baseline in the postprandial state; however, this effect was no longer significant when adjusted for the increase in MAP. Furthermore, FA composition (SFA-rich meal compared with MUFA-rich meal) had no influence on PWV_c–f_ (m/s) (Table 5).

The only study measuring PWV (m/s) in subjects with increased CVD risk factors reported no influence of FA composition (control meal compared with EPA-rich meal compared with DHA-rich meal) on postprandial PWV_c–f_ (m/s) [57] (Table 6).

## Discussion

In this review, we aimed to summarize and analyze the existing evidence on the impact of dietary fat dose and FA composition on vascular function in metabolically healthy adults and individuals with increased CVD risk, measured by postprandial changes in FMD, PWV, and AIx. We specifically focused on studies that fed commercially available foods.

### The impact of fat dose on vascular function

Studies showed that after an overnight fast of 10.0–12.5 h, a further extension of the fasting period did not affect FMD [45,46] or AIx [48]. By contrast, consumption of an HFM (breakfast or lunch) resulted in a reduction in FMD [45,46] and AIx [48] from baseline. The energy and fat content of the HFMs were comparable between studies (TABLE 2, TABLE 4).

Six trials compared the impact of HFMs and LFMs on vascular function [[40], [41], [42], [43], [44],^47^], providing inconsistent results (TABLE 2, TABLE 3, TABLE 4). Bae et al. [40] and Benson et al. [42] reported that the HFM but not the LFM affected FMD in healthy adults, resulting in a significant group difference between meals. Both trials demonstrated strong methodical quality by feeding isoenergetic meals with a strongly varying fat content (53.4 g compared with 3 g; 1 g/kg BW compared with 0.04 g/kg BW). This observation agrees with Jackson et al. [64] who report that compared with meals containing <10 g fat, meals with a higher fat content (50–105 g) impair vascular reactivity. In a recent review, Zhao et al. [65] described 3 main mechanisms by which postprandial lipemia triggers endothelial dysfunction and atherosclerosis. First, postprandial increases in TGs and TG-rich lipoproteins result in direct damage to endothelial function; this is closely linked to an imbalance in vasodilator and vasoconstrictor factors. The vasodilator decrease mainly results from decreased NO and increased oxidative stress. The second factor impairing vascular function is increased oxidative stress and decreased antioxidant capacity induced by postprandial lipemia. During postprandial lipemia, the antioxidant enzymes glutathione peroxidase and superoxide dismutase decrease, whereas the excretion of oxidative stress markers 8-external prostaglandin F2 and free 8-iso-prostaglandin F_2α_ increases; production of reactive oxygen species is also intensified during the postprandial state. Third, consumption of an HFM induces transient, low-grade inflammation with impairment of the endothelial barrier. In this process, proinflammatory genes are upregulated in endothelial cells, leukocyte activation marker expression is increased, and the proinflammatory complement system is involved [65]. Considering that, in the above-mentioned studies, the LFMs had no influence on FMD; these LFMs may not have contained enough fat (3 g, 0.04 g/kg BW) to trigger sufficient postprandial lipemia with subsequent endothelial dysfunction [40,42]. This assumption is supported by the fact that in both studies, only consumption of the HFMs but not of the LFMs resulted in a significant increase in TGs compared with baseline values.

Consistent with the findings of Bae et al. [40] and Benson et al. [42], Esser et al. [43] also observed an effect of an HFM (95 g fat) on vascular function in healthy adults, which resulted in significantly lower postprandial FMD and AIx compared with baseline values (Table 2). One mechanism by which an HFM may induce a postprandial AIx reduction is a decrease in central systolic and diastolic blood pressure following meal intake, caused by transient relaxation of arterial smooth muscles in the general circulation [66]. However, contrary to Bae et al. [40] and Benson et al. [42], Esser et al. [43] also reported a significant reduction in FMD and AIx from baseline subsequent to the LFM (14.5 g fat), without significant group differences between the HFM and LFM. The significant decrease in FMD 3 h after consumption of the LFM is surprising, especially given the results from Williams et al. [47], where a similar LFM (18.4 g fat) exerted no influence on FMD in healthy adults.

In addition to Williams et al. [47], 2 further studies detected no effect of either the HFM or the LFM on vascular function determined by FMD [41,44]. There are several reasons to explain the lack of change in FMD subsequent to the consumption of an HFM. For example, certain factors, such as physical activity determine an individual’s capacity to tolerate acute triggers that impair vascular function (e.g., an HFM) [67]. Combined with small samples sizes (often only ∼10 participants), low susceptibility to fat-induced modulation of vascular function might have prevented alterations in endothelial function following HFM consumption. Furthermore, short postprandial periods (e.g., 2 h), a small number of measurement time points (e.g., baseline and only 1 postprandial measurement), large time intervals between measurements (e.g., 4 h) may have meant that significant effects on vascular function were not detected in the postprandial period. Regarding the fact that an increase in TGs was observed during the postprandial period but no effect on vascular function was detected, Williams et al. [47] and Poitras et al. [44] noted that a rise in TGs may not consistently impair endothelial function. However, in 3 studies, the postprandial increase in TGs was correlated with the decrease in FMD assessed 2 h postprandially [40,41,46].

### The impact of FA composition on vascular function

Most of the studies that investigated the impact of FA composition on AIx showed no differential effect of meal FA composition [49,50,56] (TABLE 5, TABLE 6). However, 2 studies showed a beneficial effect of a meal enriched with DHA [57] or EPA and DHA [55] on vascular function assessed by AIx. The favorable effect of marine n–3 PUFAs (EPA/DHA) on the endothelium is widely described, including a reduction of proatherogenic and prothrombotic factors (e.g., reduced expression of endothelial adhesion molecules and proinflammatory cytokines) [68,69].

The data on the impact of FA composition on FMD is highly inconsistent (TABLE 5, TABLE 6, TABLE 7). Six studies reported no postprandial change in FMD after consumption of meals with varying FA compositions in healthy adults [53,54,63] or adults with CVD risk factors [58,59,61]. However, Rudolph et al. [52] reported that several burger meals resulted in a decrease in FMD compared with baseline, but without significant group differences. Nicholls et al. [51] found no significant group differences in FMD change from baseline after consumption of meals with 2 different fat sources; in this study, only the SFA-rich meal (coconut oil) and not the PUFA-rich meal (safflower oil) led to a significant reduction in FMD from baseline. Mechanistic studies suggest differential effects of FAs on vascular function at the molecular level. Although in human aortic endothelial cells, incubation with oleic acid promoted signal transduction via the PI3K/Akt/endothelial nitric oxide synthase (eNOS) pathway [70], palmitic acid inhibited the Akt/eNOS pathway in human umbilical vein endothelial cells, leading to a decrease in NO production by inhibition of eNOS activity [71]. On the level of FA classes, incubation of human aortic endothelial cells with SFAs and n–3 PUFAs resulted in greater downregulation of the PI3K/Akt pathway than with SFAs alone [70]. The assumption that the dietary FA composition acts as a modulator of vascular function is supported by 4 of the included studies, all of which reported a differential effect of meal FA composition on postprandial FMD [49,60,62,63]. Berry et al. [49] observed that in healthy adults, a MUFA-rich meal (high-oleic sunflower oil) led to a significantly greater postprandial reduction in FMD than the SFA-rich meal (shea butter blend). Meanwhile, Cutruzzolà et al. [63] reported that in T1DM subjects, the SFA-rich meal (butter) resulted in a significantly lower FMD than the MUFA-rich meal (olive oil). Cortés et al. [62] also fed a MUFA-rich meal enriched with olive oil, but compared the effects on FMD with those of a walnut-rich meal, reporting a decrease in FMD from baseline after an olive oil-rich meal in both healthy and hypercholesterolemic adults. Furthermore, FMD in healthy adults remained unchanged from baseline after the walnut meal, whereas FMD increased in hypercholesterolemic subjects. By showing an increase in FMD from baseline when enriching a MUFA-rich meal with ALA or EPA and DHA, West et al. [60] provided evidence for a favorable effect of n–3 PUFAs on vascular function in T2DM subjects with high fasting TGs.

### The role of health status

One study that focused on the impact of fat dose on vascular function included healthy, lean adults and adults with obesity or T2DM (Table 4) [48]. Lean subjects and those with T2DM had a higher AIx incremental AUC after consuming an HFM than obese participants. In addition, T2DM subjects showed a delay in time to return to baseline AIx values than lean individuals. Besides, in 2 different trials, Bae et al. [40,41] used the same HFM and LFM in healthy adults and CAD patients. Although in healthy adults, consumption of the HFM resulted in a significantly lower FMD than the LFM, no effect was observed in CAD subjects (TABLE 2, TABLE 3).

Concerning FA composition, 2 studies included healthy subjects and adults with hypercholesterolemia [62] and T1DM [63] (Table 7). Cortés et al. [62] reported that only in hypercholesterolemic patients but not in healthy adults, a walnut meal led to an increase in FMD. Likewise, in the study of Cutruzzolà et al. [63], FMD was unaffected by meals enriched with butter or olive oil in healthy adults, whereas in subjects with T1DM, FMD postprandially increased following the olive oil meal compared with the butter meal. In addition, West et al. [60] investigated patients with T2DM, with and without high fasting TGs. Only in subjects with high fasting TGs did enrichment of a MUFA-containing meal with ALA or EPA and DHA result in a postprandial increase in FMD (Table 6).

### Strengths and limitations

To our knowledge, this is the first review to systematically investigate the effects of fat dose and FA composition on various subclinical markers of atherosclerosis in healthy adults and CVD risk patients. The inclusion of 3 diagnostic parameters of vascular function (FMD, PWV, and AIx) enabled us to evaluate both vascular reactivity and arterial stiffness. In addition, we increased the practical application of our findings by using a food-based approach, focusing on fat sources and their FA composition from whole foods. Nevertheless, because of our focus on fat dose and FA composition, a possible influence of other meal characteristics (e.g., content of energy, carbohydrates, and antioxidants) on vascular function that might contribute to the heterogeneity of study results was not considered. The high heterogeneity of the study results was certainly also influenced by the broad range of fat amount administered via the LFMs (0–18.4 g) and HFMs (29–95 g). Because of the high variation of meal compositions (e.g., energy content and fat source), study protocols (e.g., time points of measurements and time period of protocols), and study populations and comparisons, we did not perform a meta-analysis. To obtain convincing results and more consistent evidence on the effects of meal composition on vascular function, standardization of postprandial protocols is required (Table 8). To provide a more comprehensive view of the influence of meal composition on vascular function, in addition to the diagnostic parameters presented in this review, other target systems such as inflammatory processes associated with the postprandial state and postprandial endothelial activation should be investigated. Because vascular function is multifactorially determined, and atherosclerotic processes are too complex to be reduced to just a few mechanisms (e.g., atherosclerosis caused by HFM intake), in addition to analyzing the acute influence of meal intake, other elements (e.g., habitual diet and psychologic influences) should be considered in the research on precipitating and protective factors on endothelial dysfunction and atherosclerosis.

**TABLE 8:** Recommendations for the design of future postprandial studies on vascular function

Recommendations	
Subject group	• Adult participants (≥18 y)
Study design	• Randomized • Crossover • Blinded (at least single-blinded, if possible) • Wash-out phase of 1–2 wk
Pre-intervention days	• Avoidance of intense physical activity and alcohol consumption • Overnight fast (last meal ≥10 h before first measurement)
Meal characteristics	• Breakfast • Preparation with natural, commercially available foods (e.g., pasta, bread, plant oils, and dairy products) • Characterized nutrient profile (energy content; total protein, carbohydrates, and fat; SFA, MUFA, and PUFA; dietary fiber; other nutrients, if applicable)
Fat dose	• ≥50 g/meal • Given as absolute dose or relative to body mass
Meal consumption	• Consumption within a standardized time period (e.g., 20 min) • Consumption under supervision of study personnel
Standardization before examination of vascular function	• Sufficiently long resting time in supine position (e.g., 10 min) • Rest in a quiet and climatized room • Protocol starting at the same time (an effect of circadian rhythm has been described) • No consumption of coffee or tea on the day of the examination • Standardized intake of vasoactive drugs (e.g., all vasoactive drugs should not be taken the evening before or on the day of the examination)
Measurement of vascular function	• Measurement of FMD (vascular response to hyperemia) or of PVW and AIx (arterial stiffness) • Performed by trained and blinded personnel

Abbreviations: AIx, augmentation index; FMD, flow-mediated dilation; PWV, pulse wave velocity.

## Conclusions

This review revealed 3 main findings. First, evidence suggests that meal consumption results in decreases in FMD and AIx; specifically, higher fat doses appear to impair vascular reactivity measured by FMD more strongly than lower fat doses. Second, concerning FA composition, most studies indicate no clinically relevant or contradictory effects on subclinical atherosclerosis markers (FMD, PWV, and AIx). One exception might be marine n–3 PUFAs (EPA and DHA), as data from 3 studies suggest a beneficial effect on acute vascular function. Third, some studies found differences in the vascular response to meals with varying fat doses or FA composition between metabolically healthy subjects and subjects with CVD risk factors, but based on the analyzed literature with highly heterogenic populations, the specific effects could not be deduced. Our current findings of the impact of meal total fat content and FA composition on postprandial vascular function assessed by FMD, PWV, and AIx are based on meal studies in which a variety of fat sources (e.g., virgin or refined vegetable oils, milk fat, and butter) was used in different amounts and meal recipes. At this time, it cannot be concluded which fat source in which amount and meal composition has beneficial effects on postprandial vascular function.

### Further directions

To enhance the meaningfulness of systematic reviews and to allow valid meta-analyses concerning the effects of meal composition on vascular function, we strongly recommend standardization of postprandial protocols. In Table 8, we provide an overview of fundamental aspects concerning study design. The recommendations mainly refer to the test meal and postprandial period. Meals should be fed as breakfast after an overnight fast (≥10 h). To enhance practical applications, and possibly derive subsequent dietary recommendations, the use of commercially available foods is advisable. With respect to the assumed connection between postprandial lipemia and alterations in vascular function, we recommend a fat amount of ≥50 g/meal to induce reliable lipemia. To allow synthesis and comparison of data from different studies, nutrient and FA profiles should be characterized. Finally, noninvasive measurement of vascular function should be performed after a resting period in a supine position by trained personnel.

## Author contributions

The authors’ responsibilities were as follows – HFK, SE: literature search, study selection and evaluation; HFK: preparation of the first draft of the manuscript; HFK, SE: finalization of the manuscript in close collaboration; and both authors: read and approved the final manuscript and declare responsibility for its final content.

## Conflict of interest

The authors report no conflicts of interest.

## Funding

This work was supported by the Open Access Publication Fund of the University of Bonn.

## Data availability

All data generated or analyzed during this study are included in this published article.
